# Scalp Block for Awake Craniotomy in a Patient With a Frontal Bone Mass: A Case Report

**DOI:** 10.5812/kowsar.22287523.3608

**Published:** 2012-01-01

**Authors:** Hamid Reza Amiri, Marjan Kouhnavard, Saeid Safari

**Affiliations:** 1 Department of Brain and Spinal Injury Research Centre (BASIR), Tehran University of Medical Sciences (TUMS), Tehran, Iran; 2 Department of Anesthesiology, Tehran University of Medical Sciences (TUMS), Tehran, Iran

**Keywords:** Scalp, Frontal Bone, Craniotomy

## Abstract

“Anesthesia” for awake craniotomy is a unique clinical condition that requires the anesthesiologist to provide changing states of sedation and analgesia, to ensure optimal patient comfort without interfering with electrophysiologic monitoring and patient cooperation, and also to manipulate cerebral and systemic hemodynamics while guaranteeing adequate ventilation and patency of airways. Awake craniotomy is not as popular in developing countries as in European countries. This might be due to the lack of information regarding awake craniotomy and its benefits among the neurosurgeons and anesthetists in developing countries. From the economic perspective, this procedure may decrease resource utilization by reducing the use of invasive monitoring, the duration of the operation, and the length of postoperative hospital stay. All these reasons also favor its use in the developing world, where the availability of resources still remains a challenge. In this case report we presented a successful awake craniotomy in patient with a frontal bone mass.

## 1. Introduction

Awake craniotomy has been performed since prehistoric times. Although the reasons for the trepanations remain unclear, numerous evidences obtained from studies of ancient skulls have shown that subjects have indeed survived the procedures (1). From Roger of Salerno to Cushing, the surgical techniques have developed over the centuries. After the introduction of general anesthesia, awake craniotomy is now the preferred approach for functional neurosurgery (including deep-brain stimulation for the treatment of Parkinson’s disease and, more recently, for the treatment of various other conditions, including obesity and severe obsessive compulsive disorders) although it is also used routinely and non-selectively for non-functional surgery (2). It is also indicated for epilepsy surgery and for neurosurgical procedures requiring intraoperative monitoring of speech and motor functions to localize the area of surgical interest (such as the resection or biopsy of brain tumors in eloquent areas) (3-5). Awake craniotomy enables the reduction of the adverse effects of general anesthesia (6); early postoperative neurological evaluation; speedy recovery; minimization of hospital stay (7), and consequently, resource utilization, which is particularly beneficial in developing countries (8, 9). To our knowledge, the use of awake craniotomy in developing countries is limited despite the abovementioned advantages. Reports on this procedure from hospitals and institutions in Asia are scattered and are mainly from India, China, Japan, Indonesia, and Thailand (10, 11). Here, we present a case of awake craniotomy performed for excision biopsy of a cystic mass in the right supraorbital region with no intra- or postoperative complications.

**Figure 1.**
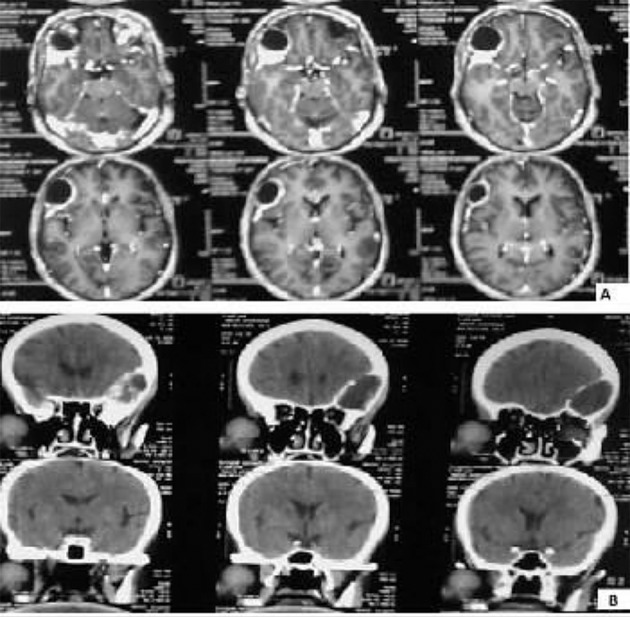
Computed Tomographic Images of the Frontal Bone Cyst Mass

## 2. Case Report

**Figure 2.**
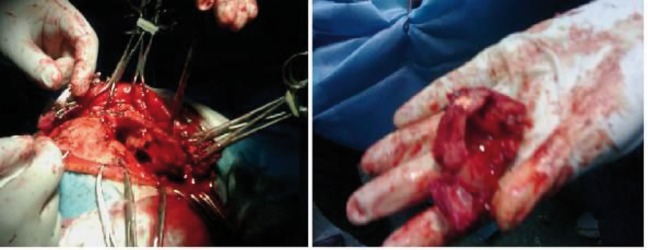
Excisional Biopsy of the Frontal Bone Cyst Mass

**Figure 3.**
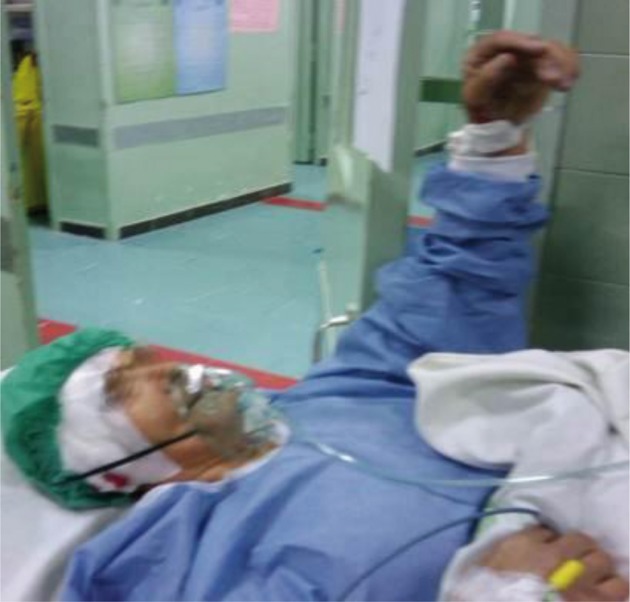
Recovery Room Just After the Operation: The Patient Was Awake and Had Stable Vital Signs.

The patient was a 61-year-old woman (weight, 67 kg) with ASA grade II and a history of chronic frontal headaches, scored about 4–5 in intensity according to the universal pain assessment tool, and decreased vision in the right eye. Brain computed tomography revealed an extradural cystic mass in the right supraorbital region (Figure 1). The patient was admitted for excision biopsy. After being explained the risks and benefits of awake craniotomy versus craniotomy under general anesthesia, the patient chose to undergo awake craniotomy and signed an informed consent. On preoperative evaluation, the findings of physical examination were normal, except for decreased vision in the right eye. Her mallampati score was 2. Routine laboratory tests, including complete blood count and tests for sodium, potassium, blood urea nitrogen, and creatinine levels, showed no abnormalities. Cardiac examination revealed that the patient could undergo the operation with moderate risk. As premedication, 2.5 mg of midazolam was administrated intravenously. Monitoring consisted of electrocardiography, pulse oximetry, invasive and non-invasive blood pressure, qualitative capnography, repetitive arterial blood gas analysis, as well as measurement of fluid intake and urinary output. Oxygen was administered via a face mask, at a rate of 5 L/min. A laryngeal mask airway, a laryngoscope, and an endotracheal tube were also prepared for use in case of significant hypoventilation or hypoxemia, or if there was a need to induce general anesthesia. At the beginning of the operation, a 100-mg injection of thiopental sodium was administered intravenously. Sedation was maintained by administration of 7–14 mL/h propofol and 10 mg diazepam in 1000 cc Ringer’s lactate at the rate of 10 mL/min. Then, scalp block was achieved in 3 quarters of the scalp as well as the left posterior part by injecting 0.17% marcaine and 0.7% lidocaine. Other drugs administered during the operation included intravenous (IV) lasix, 5 mg; 200 mL/30 min of 10% mannitol; IV bicarbonates, 3 vials; and subcutaneous lidocaine, 20 mg for arterial line placement. The patient was awake (consciously sedated) during the operation and able to speak and move her limbs on verbal command (equivalent to level 3 of the modified observer’s assessments of alertness/sedation scale). The surgery lasted for 3 h without intercurrences (3, 4). After the surgery, the patient was transferred to the recovery room, where she remained hemodynamically stable and eupneic (Figures 2 and 3). Then, she was admitted to the intensive care unit for observation during the postoperative night and discharged after 2 days.

## 3. Discussion

The idea of awake craniotomy for tumor surgery was based on its use in epilepsy surgery. Archeological studies have shown that in ancient times, thousands of years ago, patients were treated for seizures by trepanation of the skull. In “modern” times, awake craniotomy was first documented in the early 17 th century for the treatment of epilepsy (12). Classically, an awake craniotomy is indicated when the patient’s cooperation is needed for functional testing during the neurosurgical procedure. Since the introduction of awake craniotomy by Horsley at the end of the 19 th century and its subsequent use by Penfield for the surgical treatment of intractable epilepsy (13), its indications have increased and can be classified into 4 categories (14). The first category includes the procedures necessitating electrocorticographic mapping or precise electrophysiological recordings, without any interference from the effects of anesthesia; examples are epilepsy surgery and deep brain stimulation for Parkinson’s disease (15). The second category consists of resections of lesions located close to or within functionally essential motor, cognitive, or sensory cortical areas. The third category comprises procedures necessitating the obliteration or resection of vascular lesions that are essential for the vascularization of functionally important territories. Finally, the fourth category has no functional goal and encompasses minor intracranial procedures, which allows for faster recovery and earlier discharge (16). “Anesthesia” for awake craniotomy is a unique clinical condition that requires the anesthesiologist to provide changing states of sedation and analgesia, to ensure optimal patient comfort without interfering with electrophysiologic monitoring and patient cooperation, and also to manipulate cerebral and systemic hemodynamics while guaranteeing adequate ventilation and patency of airways (17). Many techniques are used to provide “anesthesia” for awake neurosurgery, depending on the institutional protocols or patients’ needs (18). Adequate local anesthesia, aimed at blocking the sensory branches of the trigeminal nerve, is essential to provide “anesthesia” for awake neurosurgery. Scalp block with the use of a local anesthetic provides reversible regional loss of sensation and reduces pain perception and global energy expenditure (19). When the preparation for the procedure is adequate, complications are rare. The most frequent intraoperative complications are obstructive apnea, nausea and vomiting, seizures, and lack of patient cooperation. Other complications are episodes of high blood pressure, bradycardia associated with a trigeminocardiac reflex, and air embolism. These complications may compromise success and necessitate conversion to general anesthesia. Experienced anesthesiologists are less prone to encounter complications than inexperienced ones (20). The avoidance of general anesthesia and its associated invasive monitoring could potentially decrease postoperative medical morbidity and shorten the length of hospital stay; therefore, recently, this technique has actually been used for outpatient craniotomy for brain tumor resection (8). Notably, one small randomized study reported higher blood loss, more neurologic deficit, and less degree of cytoreduction in patients undergoing awake craniotomy for tumors in the eloquent cortex (21). However, in a prospective study, Sackko et al. showed that patients with supratentorial lesions in proximity to the eloquent cortex had better neurologic outcome and maximal tumor removal with awake craniotomy than surgery under general anesthesia. Therefore, awake craniotomy provides a feasible alternative to craniotomy under general anesthesia. Studies comparing these 2 anesthetic options are rare in the literature (22). In the late 1990s, several case reports of awake craniotomy were published from Asia, although most of the publications were in local neurosurgery journals. Kumabe et al., in 1997, reported the case of a young man with a brain tumor in the right motor cortex who was operated under propofol anesthesia, at Tohoku University Hospital, Sendai, Japan (10). Subsequently, scattered reports on awake craniotomy have been published from other hospitals and institutions in Asia. These reports are mainly from India, China, Thailand, and Indonesia (10, 11). To our knowledge, despite its advantages, awake craniotomy is not as popular in developing countries as in European countries. This might be due to the lack of information regarding awake craniotomy and its benefits among the neurosurgeons and anesthetists in Asian countries. From the economic perspective, this procedure may decrease resource utilization by reducing the use of invasive monitoring, the duration of the operation, and the length of postoperative hospital stay (2, 8, 9). All these reasons also favor its use in the developing world, where the availability of resources still remains a challenge. Awake craniotomy appears to be well tolerated by patients and perhaps even allows them a greater measure of control and involvement in their care (9). Wrede et al. assessed patients’ acceptance of awake craniotomy objectively by using the PPP33 questionnaire and reported good tolerance for awake craniotomy in the treatment of brain tumors in or close to eloquent areas (23). There may be cultural differences across the globe in patients’ acceptance of this procedure; however, if neurosurgeons and anesthetists are willing to embrace it, they will gain confidence, which is transferred to their patients. We believe that awake craniotomy could be used more frequently and broadly by neurosurgeons to the benefit of their patients in all parts of the globe and to minimize the financial burden on health care systems, especially in developing countries.

## References

[1] Parry TW, JL. S (1936). Discovery of skull with surgical holing at Tell Duwei Palestine..

[2] Serletis D, Bernstein M (2007). Prospective study of awake craniotomy used routinely and nonselectively for supratentorial tumors.. J Neurosurg..

[3] Sanai N, Mirzadeh Z, Berger MS (2008). Functional outcome after language mapping for glioma resection.. N Engl J Med..

[4] Lega BC, Wilfong AA, Goldsmith IL, Verma A, Yoshor D (2009). Cortical resection tailored to awake, intraoperative ictal recordings and motor mapping in the treatment of intractable epilepsia partialis continua: technical case report.. Neurosurgery..

[5] Imani F, Jafarian A, Hassani V, Khan ZH (2006). Propofol-alfentanil vs propofol-remifentanil for posterior spinal fusion including wake-up test.. Br J Anaesth..

[6] Manninen PH, Tan TK (2002). Postoperative nausea and vomiting after craniotomy for tumor surgery: a comparison between awake craniotomy and general anesthesia.. J Clin Anesth..

[7] Blanshard HJ, Chung F, Manninen PH, Taylor MD, Bernstein M (2001). Awake craniotomy for removal of intracranial tumor: considerations for early discharge.. Anesth Analg..

[8] Bernstein M (2001). Outpatient craniotomy for brain tumor: a pilot feasibility study in 46 patients.. Can J Neurol Sci..

[9] Taylor MD, Bernstein M (1999). Awake craniotomy with brain mapping as the routine surgical approach to treating patients with supratentorial intraaxial tumors: a prospective trial of 200 cases.. J Neurosurg..

[10] Kumabe T, Nakasato N, Sato K, Higano S, Takahashi S, Sonoda Y (1997). [Neurophysiological monitoring during surgery for astrocytoma at the motor strip with awake craniotomy].. No Shinkei Geka..

[11] Chelani R, Borges EP (2005 May 31). Awake craniotomy has an edge over general anaesthesia. Express Healthcare Management..

[12] Horrax G., Neurosurgery. (1952). An historical sketch..

[13] Conte V, Baratta P, Tomaselli P, Songa V, Magni L, Stocchetti N (2008). Awake neurosurgery: an update.. Minerva Anestesiol..

[14] Hans P, Bonhomme V (2007). Anesthetic management for neurosurgery in awake patients.. Minerva Anestesiol..

[15] Kalenka A, Schwarz A (2009). Anaesthesia and Parkinson's disease: how to manage with new therapies?. Curr Opin Anaesthesiol..

[16] Frost EA, Booij LH (2007). Anesthesia in the patient for awake craniotomy.. Curr Opin Anaesthesiol..

[17] Dinsmore J (2007). Anaesthesia for elective neurosurgery.. Br J Anaesth..

[18] Gadhinglajkar S, Sreedhar R, Abraham M (2008). Anesthesia management of awake craniotomy performed under asleep-awake-asleep technique using laryngeal mask airway: report of two cases.. Neurol India..

[19] Yoo KY, Kim TS, Jeong CW, Kim SJ, Jeong ST, Jeong SW (2009). Anaesthetic requirements and stress hormone responses in acute cord-injured patients undergoing surgery of the injured spine.. Eur J Anaesthesiol..

[20] Berkenstadt H, Perel A, Hadani M, Unofrievich I, Ram Z (2001). Monitored anesthesia care using remifentanil and propofol for awake craniotomy.. J Neurosurg Anesthesiol..

[21] Gupta DK, Chandra PS, Ojha BK, Sharma BS, Mahapatra AK, Mehta VS (2007). Awake craniotomy versus surgery under general anesthesia for resection of intrinsic lesions of eloquent cortex--a prospective randomised study.. Clin Neurol Neurosurg..

[22] Sacko O, Lauwers-Cances V, Brauge D, Sesay M, Brenner A, Roux FE (2011). Awake craniotomy vs surgery under general anesthesia for resection of supratentorial lesions.. Neurosurgery..

[23] Wrede KH, Stieglitz LH, Fiferna A, Karst M, Gerganov VM, Samii M (2011). Patient acceptance of awake craniotomy.. Clin Neurol Neurosurg..

